# Fourth Generation Cephalosporin Resistance Among *Salmonella enterica* Serovar Enteritidis Isolates in Shanghai, China Conferred by *bla*_CTX–M–55_ Harboring Plasmids

**DOI:** 10.3389/fmicb.2020.00910

**Published:** 2020-05-15

**Authors:** Ying Fu, Xuebin Xu, Lina Zhang, Zhiying Xiong, Yeben Ma, Yihuan Wei, Zhengquan Chen, Jie Bai, Ming Liao, Jianmin Zhang

**Affiliations:** ^1^National and Regional Joint Engineering Laboratory for Medicament of Zoonoses Prevention and Control, Guangdong Laboratory for Lingnan Modern Agriculture, Laboratory of Veterinary Vaccine Innovation of the Ministry of Agriculture, Key Laboratory of Zoonosis Prevention and Control of Guangdong Province, College of Veterinary Medicine, South China Agricultural University, Guangzhou, China; ^2^Shanghai Municipal Center for Disease Control and Prevention, Shanghai, China

**Keywords:** antimicrobial susceptibility, cefepime-resistant *Salmonella* Enteritidis, extended-spectrum β-lactamase genes, pulsed-field gel electrophoresis, transconjugants

## Abstract

In this study, we investigated the pattern of antimicrobial resistance in *Salmonella* enterica serotype Enteritidis isolates in Shanghai, China from 2005 to 2014. We found the first isolates with resistance to the fourth-generation cephalosporin cefepime starting in 2010. Furthermore, we analyzed the epidemic characteristics and mechanisms of underlying cefepime resistance in *S.* Enteritidis isolates found from 2010. In total, 38 of 2,914 (1.30%) isolates were identified as cefepime-resistant *S.* Enteritidis (CRSE) isolates by Kirby–Bauer disk diffusion. Two isolates were from animal derived food sources; 36 isolates were from fecal samples of human patients with salmonellosis. Antimicrobial susceptibility testing using the agar dilution method revealed that all CRSE isolates showed additional resistances at least to ceftazidime, cefotaxime, and ampicillin. Additionally, pulsed-field gel electrophoresis (PFGE) profiles indicated that 89.47% of CRSE isolates also displayed similar PFGE patterns. Five types of β-lactamase genes, *bla*_CTX–M_ (100.00%, 38/38), *bla*_SHV_ (65.79%, 25/38), *bla*_TEM_ (52.63%, 20/38), *bla*_ACC_ (18.42%, 7/38), and *bla*_PSE_ (5.26%, 2/38) were detected by PCR and sequencing. Among *bla*_CTX–M_ genes, *bla*_CTX–M–55_ was the dominant type (84.21%, 32/38). Conjugation and transformation experiments along with plasmid replicon typing revealed that *bla*_CTX–M–55_ was located on plasmids of various replicon types with sizes ranging from 76.8 to 138.9 kb. Plasmid sequence analysis also showed that the *bla*_CTX–M–55_ gene was mobilized mainly by the IS*Ecp1*-*bla*_CTX–M–55_-ORF477 transposition unit and had its own IS*Ecp1*-based promoter, which accelerated the expression and transmission of *bla*_CTX–M–55_. Analysis of whole genome sequences (Illumina) of one selected transformant SH12G706-C showed high similarity of the *bla*_CTX–M–55_ carrying plasmid with the IncI1 plasmid backbone p628-CTX-M of *Klebsiella pneumoniae* detected in 2010 in China. The present study demonstrated that the *bla*_CTX–M–55_ gene mobilized by IS*Ecp1*- *bla*_CTX–M–55_-ORF477 was the main feature shared by CRSE isolates and seems to play an important role for transmission of cefepime resistance. The number of CRSE isolates is rising annually, and the strong dissemination ability of IS*Ecp1*-*bla*_CTX–M–55_-ORF477-harboring plasmids among different species represents an important threat to the therapeutic effectiveness of cefepime.

## Introduction

*Salmonella* is one of the most common foodborne pathogens worldwide (Tabu et al., 2012). Gastroenteritis caused in both human and animals by non-typhoid *Salmonella* (NTS) has become a global public health concern. To date, more than 2,500 *Salmonella* serotypes have been identified (Yang et al., 2010), and *S.* Enteritidis is the predominant serotype in many countries (Galanis et al., 2006; Matheson et al., 2010; Zhang et al., 2014a). According to a report by Chinese Centers for Disease Control (CDC), around 792 people died as a result of *S.* Enteritidis infection annually (Ren et al., 2016).

In general, compared to other NTS serotypes, such as *S.* Typhimurium or *S.* Derby, *S.* Enteritidis is usually self-limiting and shows susceptibility to most clinical first-line medicines (Ke et al., 2014). But antibiotics are needed for complicated cases and are used if the infection spreads or is highly likely to spread from the intestines to the bloodstream and other organs. Third generation cephalosporins have excellent curative effects, but with the abuse and inappropriate use of these antibiotics, an increasing number of *S*. Enteritidis strains are nowadays resistant, mainly caused by production of extended-spectrum beta-lactamases (ESBLs) (Tenover, 2006). This phenomenon represents an ever-increasing threat to public health and a challenge that must be met by the development of alternative therapeutic strategies. Cefepime, which is the first fourth generation cephalosporin to be approved for use in China, has been reported to show low toxicity and high activity against *Enterobacteriaceae* that resistant to third-generation cephalosporins (Chapman and Perry, 2003; Yahav et al., 2007). There are some reports stated that cefepime should be used with caution for serious ESBL infections based on its failure on some clinical experiments (Labombardi et al., 2006; Wang et al., 2016; Tamma and Rodriguez-Bano, 2017). But it can be considered for treatment if the MIC ≤ 1 μg/mL and meanwhile taken the patient’s health conditions into consideration, such as the severity of infection, resistance to antibiotics (Patel et al., 2019).

Despite the important role of cefepime in treating ESBL-producing *S.* Enteritidis infections, the epidemiological data in this field are scarce. Therefore, in this study, we conducted a systematic characterization of the epidemiology of *S*. Enteritidis infections in Shanghai, China from 2005 to 2014 to clarify the mechanisms underlying the development of cefepime-resistant *S*. Enteritidis.

## Materials and Methods

### Bacterial Strains

During the period from 2005 to 2014, a total of 2,914 *S.* Enteritidis isolates were collected by the Shanghai CDC from human diarrhea samples obtained from 134 hospitals and animal-derived food obtained from 123 retail markets. The *Salmonella* isolates were serotyped according to the White Kauffmann Le Minor scheme (Issenhuth-Jeanjean et al., 2014) or National Food Safety Standard-Food microbiological examination: *Salmonella* (GB 4789.4–2016) by slide agglutination, using specific O and H antisera (S&A Reagents Lab Ltd., Bangkok, Thailand). All isolates were then transported to the Key Laboratory of Zoonosis Prevention and Control of Guangdong Province in South China Agriculture University by a medium that can keep bacteria alive at room temperature for 12 months (Kemajia, Shanghai, China). The Kirby–Bauer disk diffusion method was used to select the cefepime-resistant *S.* Enteritidis (CRSE) isolates and characterize their antimicrobial susceptibility profiles. This test was performed on Mueller–Hinton agar with seventeen impregnated disks (Oxoid, United Kingdom) as follows: amoxicillin-clavulanic acid 30 μg (AMC), ampicillin 10 μg (AMP), cefotaxime 30 μg (CTX), ceftazidime 30 μg (CAZ), cefepime 5 μg (FEP), imipenem 10 μg (IPM), amikacin 30 μg (AMK), gentamicin 10 μg (GEN), streptomycin 10 μg (STR), sulfisoxazole 300 μg (SIZ), trimethoprim-sulfamethoxazole 23.75/1.2 μg (SXT), polymyxin B 300 IU (PMB), chloramphenicol 30 μg (CHL), tetracycline 30 μg (TET), nalidixic acid 30 μg (NAL), ciprofloxacin 5 μg (CIP), and ofloxacin 5 μg (OFX). The minimum inhibitory concentrations (MICs) of the selected cefepime-resistant isolates and their transconjugants to three beta-lactam antibiotics (cefotaxime, ceftazidime and cefepime) were determined by agar dilution in accordance with the standards and guidelines described by the Clinical and Laboratory Standards Institute (CLSI). *E. coli* ATCC 25922 was used as the quality control and the results were interpreted according to the CLSI standards and guidelines 2017.

### Detection of Beta-Lactamase Genes

*Salmonella* isolates showing resistance to cefepime were further subjected to screening for the presence of beta-lactamase genes (*bla*_CTX–M_, *bla*_TEM_, *bla*_SHV_, *bla*_ACC_, *bla*_OXA_, *bla*_PSE_, *bla*_VEB_, *bla*_PER_, and *bla*_GES_) by PCR assays (Kiratisin et al., 2008; Usha et al., 2008). All *bla*_CTX–M_ positive PCR products were sequenced and aligned using GenBank online BLAST software. The primers used in this study are shown in Supplementary Table S1.

### Pulsed-Field Gel Electrophoresis (PFGE)

PFGE subtyping of 38 CRSE was performed using *X*baI-digestion of genomic DNA according to the protocol recommended by the CDC (Ribot et al., 2006). The PFGE patterns were then compared using BioNumerics software (Version 5.1; Applied-Maths, Sint-Martens-Latem, Belgium).

### Conjugation and Transformation Experiments

Conjugation experiments were conducted in Luria-Bertani broth, using CRSE as donor strains and the streptomycin-resistant *E. coli* strain C600 as the recipient. Cultures of donor and recipient cells in the logarithmic phase (1 and 4 mL, respectively) were mixed and incubated overnight at 37°C without shaking. If conjugation failed the mixture was exposed to a brief pulse of a high-voltage electric field (1.8 kv). 1 mL LB broth was added to the mixture after electric shock to recover the bacteria. *E*. *coli* C600 transconjugants/transformants with ESBL genes on plasmids provided by *S*. Enteritidis were identified on the basis of the ability to generate blue bacterial colonies on CHROMagar^TM^ containing ceftazidime (2 μg/mL) plus streptomycin (3,000 μg/mL, Sigma–Aldrich Co., St Louis, MO, United States). To further verify the transconjugants/transformants, MICs of cefotaxime, ceftazidime, cefepime and the presence of *bla*_CTX–M_ were determined as described above.

### Plasmid Characterization

The incompatibility groups of plasmids harbored by transconjugants/transformants were determined by PCR-based replicon typing using previously reported primers (Carattoli et al., 2005). The sizes of bacterial plasmids carrying *bla*_CTX–M_ gene were determined by PFGE of S1 nuclease (TaKaRa Biotechnology, Dalian, China)-digested whole genomic DNA; the location of the *bla*_CTX–M_ gene was determined by Southern blot hybridization using probes for the specific detection of the DIG-labeled *bla*_CTX–M_ fragment according to the manufacturer’s instructions (DIG High Prime DNA Labeling and Detection Starter Kit I, Roche Applied Science, Mannheim, Germany) (Yang et al., 2012). The genetic context of *bla*_CTX–M_ was determined by PCR using previously described primers and conditions (Pan et al., 2013).

### Sequencing and Analysis of a *bla*_CTX–M–55_-Carrying Plasmid

The plasmid that transferred from the *bla*_CTX–M–55_ positive *S.* Enteritidis isolate SH12G706 to *E. coli* C600 was extracted using the Omega Plasmid BAC/PAC DNA Kit D2156 (Omega, Bio-Tek) and then sent to GENEWIZ (Guangzhou, China) for whole genome *de novo* sequencing by using Illumina HiSeq sequencing technology. The contigs was assembled using Velvet (version 1.2.10), SSPACE (version 3.0), and GapFiller (version 1–10) software and the annotation of genes was conducted using Non-Redundant Protein, gene ontology and Kyoto encyclopedia of genes and genomes databases. The nucleotide and amino acid sequences were analyzed and compared through BLAST queries against the GenBank database. The datasets generated for this study can be found in the NCBI-Sequence Read Archive (SRA): SRR9734360.

## Results

### Prevalence of CRSE

In total, 2,914 *S.* Enteritidis isolates were obtained, of which, 222 isolates were collected in 2010, 679 isolates were collected in 2011, 547 isolates were collected in 2012, 629 isolates were collected in 2013 and 462 isolates were collected in 2014. In total, 2,566 isolates were human-derived, while 348 isolates were from animal-derived food, see detailed information in Table 1. *S.* Enteritidis could be detected all year-round, but mainly in summer and autumn. Furthermore, adults (men and women) and children were more often to be infected than infants and the elderly. CRSE first appeared in a clinical diarrhea sample in 2010 and more recently, in foodborne source in 2013. Subsequently, 38 CRSE isolates (1.3%; 38/2914) were obtained with increasing frequency year by year; 36 CRSE were from human patients (17 hospitals) and 2 from animal-derived food (chicken wings and duck breast).

**TABLE 1:** The prevalence of *Salmonella* Enteritidis isolates showing resistance to cefepime in Shanghai, China from 2005 to 2014.

Year	2005	2006	2007	2008	2009	2010	2011	2012	2013	2014
Number (%)	*n* (%)	*n* (%)	*n* (%)	*n* (%)	*n* (%)	*n* (%)	*n* (%)	*n* (%)	*n* (%)	*n* (%)
Human-derived	0 (0.00%, 0/8)	0 (0.00%, 0/62)	0 (0.00%, 0/57)	0 (0.00%, 0/125)	0 (0.00%, 0/123)	1 (0.52%, 1/192)	6 (1.01%, 6/597)	14 (2.84%, 14/492)	8 (1.42%, 8/562)	7 (1.65%, 7/425)
Animal-derived food*	0 (0.00%, 0/0)	0 (0.00%, 0/0)	0 (0.00%, 0/0)	0 (0.00%, 0/0)	0 (0.00%, 0/0)	0 (0.00%, 0/30)	0 (0.00%, 0/82)	0 (0.00%, 0/55)	1 (1.49%, 1/67)	1 (2.70%, 1/37)
Total	0 (0.00%, 0/8)	0 (0.00%, 0/62)	0 (0.00%, 0/57)	0 (0.00%, 0/125)	0 (0.00%, 0/123)	1 (0.45%, 1/222)	6 (0.88%, 6/679)	14 (2.56%, 14/547)	9 (1.43%, 9/629)	8 (1.73%, 8/462)

*^∗^Animal-derived food: chicken wings (n = 1), duck breast (n = 1).*

### Antimicrobial Susceptibility Profiles

The 38 isolates with cefepime resistance were simultaneously resistant to ceftazidime (100%), cefotaxime (100%) and ampicillin (100%) and also showed high resistance to nalidixic acid (97.37%), sulfafurazole (76.32%), streptomycin (63.16%) and trimethoprim-sulfamethoxazole (52.63%), followed by tetracycline (44.74%) and chloramphenicol (28.95%). However, the CRSE isolates were more susceptible to amoxicillin-clavulanic acid (15.79%), gentamicin (13.16%), amikacin (13.16%). They all showed intermediate susceptibility to ciprofloxacin, ofloxacin, polymyxin B and imipenem (Table 2 and Supplementary Table S2).

**TABLE 2:** Antimicrobial resistance of 38 cefepime-resistant *Salmonella* Enteritidis isolates.

Antibiotic	Percentage of drug resistance: *n* (%)		
	R	I	S
	
	*n* (%)	*n* (%)	*n* (%)
Amoxicillin-clavulanic acid	6(15.79%)	31(81.58%)	1(2.63%)
Ampicillin	38(100.00%)	0(0.00%)	0(0.00%)
Cefotaxime	38(100.00%)	0(0.00%)	0(0.00%)
Ceftazidime	38(100.00%)	0(0.00%)	0(0.00%)
Cefepime	38(100.00%)	0(0.00%)	0(0.00%)
Imipenem	0(0.00%)	38(100.00%)	0(0.00%)
Amikacin	1(2.63%)	37(97.37%)	0(0.00%)
Gentamicin	5(13.16%)	32(84.21%)	1(2.63%)
Streptomycin	24(63.16%)	12(31.58%)	2(5.26%)
Sulfisoxazole	29(76.32%)	9(23.68%)	0(0.00%)
Trimethoprim-sulfamethoxazole	20(52.63%)	18(47.37%)	0(0.00%)
Polymyxin B	0(0.00%)	38(100.00%)	0(0.00%)
Chloramphenicol	11(28.95%)	21(55.26%)	6(15.79%)
Tetracycline	17(44.74%)	21(55.26%)	0(0.00%)
Nalidixic acid	37(97.37%)	1(2.63%)	0(0.00%)
Ciprofloxacin	0(0.00%)	38(100.00%)	0(0.00%)
Ofloxacin	0(0.00%)	38(100.00%)	0(0.00%)

Isolates resistant to cefepime were 100% multidrug-resistant (MDR), with resistance to a minimum of five antibiotics and a maximum of 10 antibiotics and 6 isolates were resistant to ten different antibiotics. The spectrum of resistance types reached 19 with the following three combinations detected in higher proportions, “AMP-CTX-CAZ-FEP-STR-SIZ-SXT-CHL-TET-NAL” (13.16%, 5/38), “AMP-CTX-CAZ-FEP-STR-SIZ-TET-NAL” (13.16%, 5/38), and “AMP-CTX-CAZ-FEP-GEN”(13.16%, 5/38) (Supplementary Table S3).

### Prevalence of Beta-Lactamase Genes

The beta-lactamase-encoding genes *bla*_CTX–M_ (100.00%), *bla*_SHV_ (65.79%), *bla*_TEM_ (52.63%), *bla*_ACC_ (5.26%) and *bla*_PSE_ (5.26%) were identified. Coexistence of two or more genes in a single CRSE was very common. Sequencing of the *bla*_CTX–M_ genes revealed presence of various types: *bla*_CTX–M–55_ (84.21%, 32/38), *bla*_CTX–M–64_ (5.26%), *bla*_CTX–M–123_ (5.26%), *bla*_CTX–M–3_ (2.63%), and *bla*_CTX–M–15_ (2.63%).

#### Pulse Field Gel Electrophoresis (PFGE)

**FIGURE 1:**
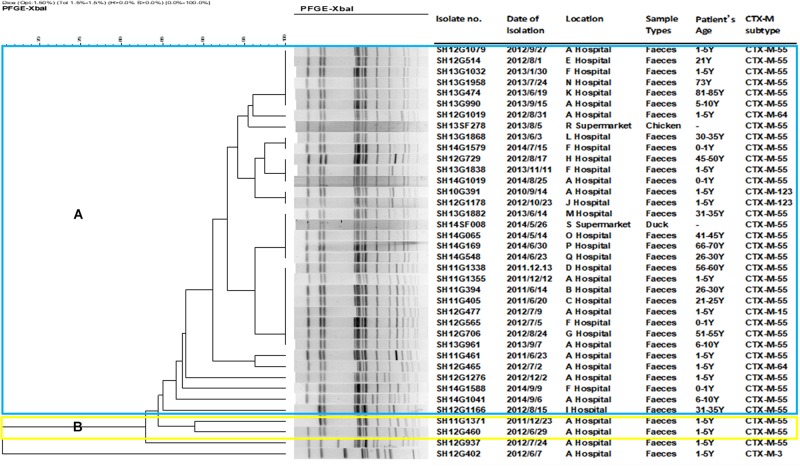
Dendrogram of pulsed-field gel electrophoresis (PFGE) profiles of 38 cefepime-resistant *Salmonella* Enteritidis isolates. The blue color represents cluster **(A)**, which includes 34 isolates; the yellow color represents cluster **(B)**, which including 2 isolates; another 2 isolates were not assigned to clusters **(A,B)**.

A total of 18 different PFGE patterns were obtained from the 38 CRSE isolates (Figure 1). The similarity ranged from 65.44 to 100%. PFGE genotypes of >85% similarity were grouped into 2 clusters and another two individual isolates. The PFGE genotype of one foodborne isolate with CTX-M-55 (SH14SF008) was identical to four CTX-M-55 producing isolates from clinical samples (SH13G1882, SH14G065, SH14G169, SH14G548). However, several isolates from different years and hospitals also shared the same PFGE genotype (Figure 1).

### Plasmid Characterization

In total, 35 transconjugants were successfully obtained from 38 CRSE isolates through the conjugation experiments; the transfer efficiency was 92.11% (35/38). Transfer of the *bla*_CTX–M_ gene alone was detected in 33 of 35 transconjugants; two transconjugants harbored combinations (*bla*_CTX–M–3_ + *bla*_TEM_; *bla*_CTX–M–55_ + *bla*_SHV_) (Supplementary Figures S1–S3). *bla*_CTX–M–55_ gene could be found on 76.8 kb IncI1 (*n* = 9), 76.8 kb IncFrepB (*n* = 5), 104.5 kb IncFrepB (*n* = 1), 138.9 kb IncFrepB (*n* = 1), 138.9 kb IncFrepB/N (*n* = 1), 104.5 kb IncFrepB/FIIA (*n* = 1), 76.8 kb IncFIIA/FIIB/FrepB (*n* = 3), 104.5 kb IncFIIA/FIIB/FrepB (*n* = 4), 104.5∼138.9 kb IncFIIA/FIIB/FrepB (*n* = 1), 138.9 kb IncFIIA/FIIB/FrepB (*n* = 1) and >138.9 kb IncN/FIIB/FrepB (*n* = 1) plasmids; while *bla*_CTX–M–3_ and *bla*_TEM_ could be observed on 76.8 kb IncL/M plasmid (*n* = 1); *bla*_CTX–M–55_ and *bla*_SHV_ could be observed on 104.5 kb IncFrepB/FIIA plasmid (*n* = 1). Comparing to the recipient strain *E*. *coli* C600, horizontal transfer of *bla*_CTX–M_ genes resulted in an increase of MICs for cefotaxime (from 128 to 2,048 mg/L), ceftazidime (from 4 to 64 mg/L) and cefepime (from 16 to 64 mg/L) (Table 3). The simultaneous presence of two or three plasmid replicons in one *bla*_CTX–M–55_-carrying plasmid was observed in this study. Southern blot hybridization suggested that the size of the *bla*_CTX–M_ gene carrying plasmids varied between 76.8 and 138.9 kb (Supplementary Figures S1–S3). In genetic context detection tests, the insertion sequence IS*Ecp1* or IS*903* appeared frequently upstream and downstream of *bla*_CTX–M_, functioning as a mobile element in the process of horizontal transmission (Yi et al., 2010). Totally, 45.7% (16/35) transconjugants harbored “IS*Ecp1*-F-*bla*_CTX–M–55_-ORF477” element, which had been reported in *Shigella sonnei* strain before (Qu et al., 2014). The characteristics of the transconjugant plasmids are shown in Table 3.

**TABLE 3:** Characteristics of cefepime-resistant *Salmonella* Enteritidis donor strains and transconjugants.

Isolates	Beta-lactamase gene	No. of plasmids	Size of plasmids harboring the *bla*_CTX–M_ gene	Replicon type	Upstream of *bla*_CTX–M_	Downstream of *bla*_CTX–M_	MIC (μ g/mL)		
							CTX	CAZ	FEP
*E. coli* C600							<0.125	<0.125	<0.125
SH11G394	*bla*_CTX–M–55_, TEM, SHV	1	76.8 kb				256	32	32
SH11G394-C*	*bla*_CTX–M–55_	1	76.8 kb	I1	IS*Ecp1*	ORF-477	16	2	2
SH11G405	*bla*_CTX–M–55_, TEM	1	76.8 kb				256	32	32
SH11G405-C*	*bla*_CTX–M–55_	1	76.8 kb	I1	IS*Ecp1*	ORF-477	64	4	8
SH11G1338	*bla*_CTX–M–55_, TEM, SHV	1	76.8 kb				256	32	32
SH11G1338-C*	*bla*_CTX–M–55_	1	76.8 kb	I1	IS*Ecp1*	ORF-477	64	4	8
SH11G1355	*bla*_CTX–M–55_, TEM, SHV	1	76.8 kb				256	32	32
SH11G1355-C*	*bla*_CTX–M–55_	1	76.8 kb	I1	IS*Ecp1*	ORF-477	128	4	8
SH11G1371	*bla*_CTX–M–55_, TEM	1	76.8 kb				256	32	32
SH11G1371-C*	*bla*_CTX–M–55_	1	76.8 kb	I1	IS*Ecp1*	ORF-477	64	8	8
SH12G565	*bla*_CTX–M–55_, TEM	1	76.8 kb				256	32	32
SH12G565-C*	*bla*_CTX–M–55_	1	76.8 kb	I1	IS*Ecp1*	ORF-477	32	8	4
SH12G706	*bla*_CTX–M–55_, TEM	1	76.8 kb				256	32	32
SH12G706-C*	*bla*_CTX–M–55_	1	76.8 kb	I1	IS*Ecp1*	ORF-477	32	4	4
SH13G961	*bla*_CTX–M–55_	1	76.8 kb				256	32	32
SH13G961-C*	*bla*_CTX–M–55_	1	76.8 kb	I1	IS*Ecp1*	ORF-477	128	4	8
SH12G460	*bla*_CTX–M–55_, TEM, SHV	2	54.7 kb, 76.8 kb				>512	64	32
SH12G460-C*	*bla*_CTX–M–55_	1	76.8 kb	FIIA, FIIB, FrepB		ORF-477	32	4	4
SH12G514	*bla*_CTX–M–55_	1	104.5 kb				256	32	32
SH12G514-C*	*bla*_CTX–M–55_	1	104.5 kb	FrepB		ORF-477	256	4	8
SH12G729	*bla*_CTX–M–55_, TEM, SHV, PSE, ACC	1	76.8 kb				256	32	32
SH12G729-C*	*bla*_CTX–M–55_	1	76.8 kb	FrepB		ORF-477	32	4	2
SH12G1079	*bla*_CTX–M–55_, SHV	1	104.5 kb				256	32	32
SH12G1079-C*	*bla*_CTX–M–55_, SHV	1	104.5 kb	FIIA, FrepB		ORF-477	16	4	2
SH12G1166	*bla*_CTX–M–55_, TEM, SHV	2	104.5 kb, >138.9 kb				128	16	32
SH12G1166-C*	*bla*_CTX–M–55_	1	>138.9 kb	N, FIIB, FrepB		ORF-477	128	4	8
SH13G474	*bla*_CTX–M–55_, TEM, SHV	2	54.7 kb, 76.8 kb				256	32	32
SH13G474-C*	*bla*_CTX–M–55_	1	76.8 kb	FIIA, FIIB, FrepB		ORF-477	64	4	4
SH13G990	*bla*_CTX–M–55_, SHV	2	54.7 kb, 104.5 kb				256	32	32
SH13G990-C*	*bla*_CTX–M–55_	1	104.5 kb	FIIA, FIIB, FrepB		ORF-477	128	4	4
SH13G1032	*bla*_CTX–M–55_, SHV	1	76.8 kb				256	32	32
SH13G1032-C*	*bla*_CTX–M–55_	1	76.8 kb	FIIA, FIIB, FrepB		ORF-477	64	4	4
SH13G1838	*bla*_CTX–M–55_, SHV	1	138.9 kb				256	32	32
SH13G1838-C*	*bla*_CTX–M–55_	1	138.9 kb	N, FrepB		ORF-477	128	4	4
SH13G1868	*bla*_CTX–M–55_, SHV	1	76.8 kb				256	32	32
SH13G1868-C*	*bla*_CTX–M–55_	1	76.8 kb	FrepB		ORF-477	16	0.5	2
SH13G1882	*bla*_CTX–M–55_	1	76.8 kb				256	32	16
SH13G1882-C*	*bla*_CTX–M–55_	1	76.8 kb	FrepB		ORF-477	64	4	4
SH13G1958	*bla*_CTX–M–55_, SHV, ACC	2	54.7 kb, 138.9 kb				256	32	32
SH13G1958-C*	*bla*_CTX–M–55_	1	138.9 kb	FIIA, FIIB, FrepB		ORF-477	64	4	4
SH14G065	*bla*_CTX–M–55_, SHV, ACC	1	76.8 kb				256	32	32
SH14G065-C*	*bla*_CTX–M–55_	1	76.8 kb	FrepB		ORF-477	128	4	4
SH14G169	*bla*_CTX–M–55_, SHV, ACC	2	54.7 kb, 104.5 kb				256	32	32
SH14G169-C*	*bla*_CTX–M–55_	1	104.5 kb	FIIA, FIIB, FrepB		ORF-477	128	4	4
SH14G548	*bla*_CTX–M–55_, SHV, ACC	2	54.7 kb, 76.8 kb				256	32	32
SH14G548-C*	*bla*_CTX–M–55_	1	76.8 kb	FIIA, FIIB, FrepB		ORF-477	128	4	8
SH14G1579	*bla*_CTX–M–55_	2	54.7 kb, 104.5 kb				256	32	32
SH14G1579-C*	*bla*_CTX–M–55_	1	104.5 kb	FIIA, FIIB, FrepB		ORF-477	64	4	4
SH13SF278	*bla*_CTX–M–55_, SHV	2	54.7 kb, 138.9 kb				256	32	32
SH13SF278-C*	*bla*_CTX–M–55_	1	138.9 kb	FrepB	IS*Ecp1*	ORF-477	128	8	8
SH14SF008	*bla*_CTX–M–55_, SHV	2	54.7 kb, 104.5 kb ∼ 138.9 kb				256	32	16
SH14SF008-C*	*bla*_CTX–M–55_	2	54.7 kb, 104.5 kb ∼ 138.9 kb	FIIA, FIIB, FrepB		ORF-477	128	4	2
SH12G402	*bla*_CTX–M–3_, TEM, SHV, PSE	1	76.8 kb				128	8	32
SH12G402-C*	*bla*_CTX–M–3_, TEM	1	76.8 kb	L/M	IS*Ecp1*	ORF-477	32	1	8
SH14G1019	*bla*_CTX–M–55_, TEM, SHV	2	54.7 kb, 104.5 kb				>512	512	128
SH14G1019-C*	*bla*_CTX–M–55_	1	104.5 kb	FIIA, FIIB, FrepB	IS*Ecp1*	ORF-477	128	4	8
SH14G1041	*bla*_CTX–M–55_, TEM, SHV	1	76.8 kb				>512	512	64
SH14G1041-C*	*bla*_CTX–M–55_	1	76.8 kb	FrepB	IS*Ecp1*	ORF-477	32	2	2
SH12G477	*bla*_CTX–M–15_, TEM	1	104.5 kb				256	32	32
SH12G477-C*	*bla*_CTX–M–15_	1	104.5 kb	I1	IS*Ecp1*	ORF-477	64	4	8
SH12G1178	*bla*_CTX–M–123_, TEM	1	104.5 kb ∼ 138.9 kb				512	64	32
SH12G1178-C*	*bla*_CTX–M–123_	1	104.5 kb ∼ 138.9 kb	I1	IS*Ecp1*	ORF-477	128	8	8
SH12G465	*bla*_CTX–M–64_, SHV	1	76.8 kb				>512	32	32
SH12G465-C*	*bla*_CTX–M–64_	1	76.8 kb	-	IS*Ecp1*	ORF-477	64	8	8
SH12G1019	*bla*_CTX–M–64_, TEM, SHV	1	76.8 kb				>512	32	32
SH12G1019-C*	*bla*_CTX–M–64_	1	76.8 kb	Y		ORF-477	64	8	4
SH10G391	*bla*_CTX–M–123_, TEM	1	104.5 ∼ 138.9 kb				512	64	32
SH10G391-C*	*bla*_CTX–M–123_	1	104.5 ∼ 138.9 kb	I1	IS*Ecp1*	ORF-477	128	8	8
SH12G1276	*bla*_CTX–M–55_	1	138.9 kb				128	2	32
SH12G1276-C*	*bla*_CTX–M–55_	1	138.9 kb	-	IS*Ecp1*	IS903	16	0.5	4

*^∗^Isolates labeled with the ending “-C” are transconjugants of the respective donor strains.*

### Sequence Analysis of a *bla*_CTX–M–55_ Carrying Plasmid

**FIGURE 2:**
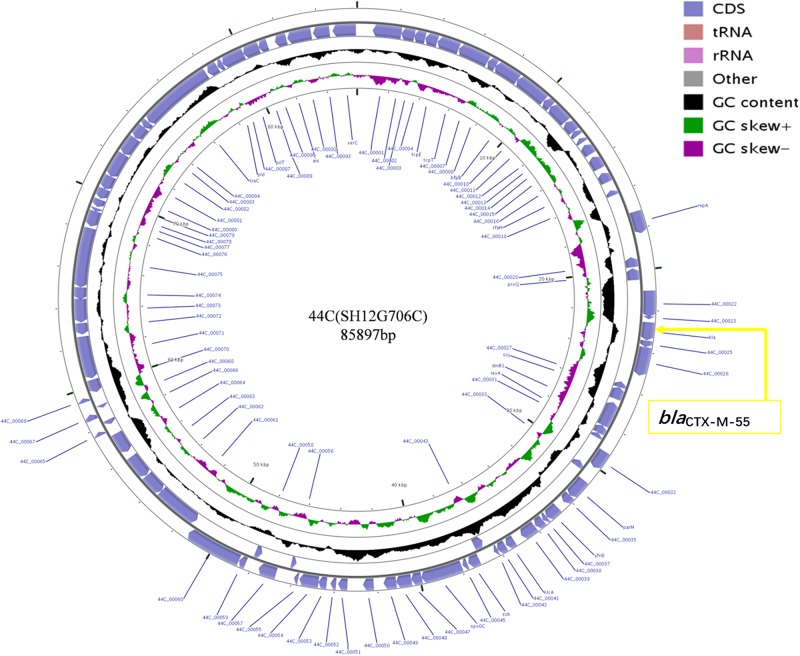
Circular map of plasmid 44C isolated from a transconjugant (donor strain *S.* Enteritidis no. SH12G706). The blue arrows represent open reading frames and their direction of transcription. The yellow box represents the location of gene *bla*_CTX–M–55_.

**FIGURE 3:**
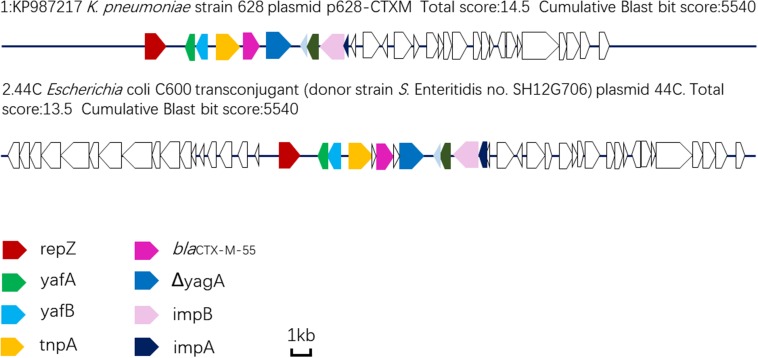
Linear comparison of DNA sequences of plasmid 44C isolated from a transconjugant (donor strain *S*. Enteritidis no. SH12G706) and plasmid p628-CTX-M from a *K*. pneumoniae isolate china, 2010.

Plasmid sequencing and assembly revealed that the plasmid transferred from the *bla*_CTX–M–55_ positive *S.* Enteritidis isolate SH12G706 to *E. coli* C600 (designated 44C) was 85,897 bp in length with an average G+C content of 49.67% and harbored a total of 95 ORFs annotated to genes associated with plasmid replication (*repA*), IncI1 conjugation transfer (*traA-Y, trbA-C, pilA-X*), plasmid SOS inhibition (*psiA, psiB*), intracellular multiplication (*icmB, icmO, icmP, dotL, dotO*), plasmid segregation (*parA, parM, soj*) and plasmid stability (*stbA, stbB*) (Figure 2). When compared with a range of plasmids that harbored *bla*_CTX–M–55_ gene, the flanking region of *bla*_CTX–M–55_ on plasmid 44C showed high similarity with plasmid p628-CTX-M (KP987217, *K. pneumoniae*, china, 2010) that had a 2,980 bp IS*Ecp1*-related element that functioned as a mobile unit (Figure 3).” Both plasmids showed the linear structure “*repZ*-*yafA*-*yafB* -*tnpA*-*bl*a_CTX–M–55_-Δ*yagA.*”

## Discussion

In the investigation period from 2005 to 2014 in Shanghai, China, *S.* Enteritidis strains showed only a slight variation in resistance to commonly used drugs, including third generation cephalosporins (Supplementary Table S4). This finding is in contrast to previous reports that other *Salmonella* serotypes (e.g., Typhimurium), exhibited an increasing tendency of resistance to at least 10 types of antibiotics during the same period (Ni et al., 2013). However, it is noteworthy that CRSE strains emerged in 2010 and their frequency is rising annually.

In recent years, cefepime ranked the leading anti-infective medicine in many hospitals in China. In order to prevent the spread of antibiotic resistance, cefepime is rarely used for animals or in breeding industry. However, our study shows that cefepime-resistant *S.* Enteritidis has also been isolated from animal-derived foodborne sources since 2013.

In this study, we analyzed 38 CRSE isolates, all of which exhibited MDR and the frequency of isolates resistant to more than five antibiotics was very high (100%). The emergence of severe drug-resistant status in China is largely related to the abuse of antibiotics as feed additives in veterinary clinical practice and the transfer of movable components carrying drug-resistant genes between isolates, which has become a common problem in both human and veterinary clinical practice (Rahmani et al., 2013). However, a relatively low resistance to amoxicillin-clavulanic acid (15.79%), gentamicin (13.16%), and amikacin (13.16%) was detected in the 38 cefepime-resistant *S*. Enteritidis isolates. They all showed intermediate susceptibility to ciprofloxacin, ofloxacin, polymyxin B and imipenem; therefore, these drugs should be used with caution. The fact also emphasizes the importance of standardization of clinical drug application and to prolong the effectiveness of new drugs. In addition, although the isolates were sensitive to aminoglycosides and fluoroquinolones, these agents should be used with caution due to the ototoxicity and adverse effects of cartilage on children.

In this study, nine kinds of β-lactamase resistance genes were detected, among them, subtype *bla*_CTX–M–55_ was predominant. Based on amino acid sequence differences, CTX-M-type ESBLs can be divided into more than five groups and hundreds of variants. CTX-M-55 belongs to the CTX-M-1 group, which was first detected in *E. coli* in Thailand in 2005 (Kiratisin et al., 2007) and first detected in *Salmonella* in 2011 (Yu et al., 2011). CTX-M-55 has only one amino acid site difference (valine to alanine) compared with CTX-M-15 (Kiratisin et al., 2007), and exhibits high hydrolytic activity to cefotaxime and ceftazidime (Dallenne et al., 2010). The prevalence of CTX-M-55-producing bacteria in China has grown significantly in recent years in both animal and human populations (Bevan et al., 2017). Data showed that CTX-M-55 is widely distributed in foodborne *E. coli* in Asia regions (Zhang et al., 2014b), which was the second most common CTX-M genotype (26.1%, 29/111) following CTX-M-14 (Zheng et al., 2012). Regarding hospital-associated *Enterobacteriaceae* infections, CTX-M-55 was found to be more common than CTX-M-15 in China (Xia et al., 2014). Also, CTX-M-55 was the most prevalent ESBL type observed in *Salmonella* isolates from livestock animals in China (Zhang et al., 2019). It is known that CTX-M-55 exists in bacteria that show resistance to second or third generation cephalosporins, and in this study, we found that CTX-M-55-producing *S.* Enteritidis was also with resistance to fourth-generation cephalosporin, cefepime. Therefore, CTX-M-55 should be considered as an important surveillance target around the world.

The *bla*_CTX–M_ genes of 35 of the 38 isolates were transferred to the recipient by conjugation. Under the influence of *bla*_CTX–M_, the transconjugants showed resistance to cephalosporins but the MICs varied. They did not reach the same MIC values as the donor bacterium, a difference that may be related to the complex mechanisms of antibiotic resistance such as efflux pumps and penicillin-binding protein in the donor bacterium.

In this study, transconjugants carrying *bla*_CTX–M_ shared different types of replicons, mainly IncI1 and InFrepB, with sizes ranging from 76.8 to 138.9 kb. The genetic context of *bla*_CTX–M_ was mainly “IS*Ecp1*-F-*bla*_CTX–M–55_-ORF477.” IS*Ecp1*, like IS*26* and IS*CR1*, is a member of the IS*1380* family. This component shares a similar structure with a promoter, which positively regulates the high-level expression of *bla*_CTX–M_ and facilitates its horizontal transmission among various species (Dhanji et al., 2011; Singh et al., 2018).

PFGE analysis showed that most CRSE displayed similar PFGE patterns (>82.90%). Notably, the pattern of one foodborne isolate (SH14SF008) was identical to that of another four isolates collected from clinical diarrhea human samples and all had the same *bla*_CTX–M–55_ gene, which suggested that we should pay attention to food consumption. Furthermore, *S*. Enteritidis isolates from different years showed 100% homology, indicating possible long-term outbreaks caused by clonal transfer of *S*. Enteritidis strains within the hospital or recurring introduction and spread of *Salmonella* in the hospital by colonized humans or contaminated food (Blahova et al., 1998). CRSE long term presence in animal breeding and especially fattening lots has been described (Ramchandani et al., 2005; Parveen et al., 2007). On the other hand, the limited discriminatory power of PFGE for several *Salmonella* serovars is known; this might falsely indicate clonal transfer. Therefore, WGS-based phylogenetic analyses are needed in future to assess the genetic relationship of the CRSE isolates in more detail.

WGS analysis of the plasmid 44C, which was from CRSE isolate of a diarrhea patient, showed that it had genes associated with plasmid replication, conjugation transfer, inhibition, intracellular multiplication, segregation and stability, functional genes that confer the ability to transfer *bla*_CTX–M–55_ and integrate into the genome of other bacteria. Moreover, compared with other *bla*_CTX–M–55_-harboring plasmids, we found that plasmid 44C showed high sequence similarity with plasmid p628-CTX-M isolated from a *K. pneumoniae* strain in October 2010 (Wang et al., 2015). In particular, both plasmids shared an IS*Ecp1*-related component in the genetic context around *bla*_CTX–M–55_, indicating that such structures may transfer from *K. pneumoniae* to *Salmonella*. Thus, it is crucial to implement precautionary measures to prevent the spread of bacteria with these mobile resistance genes in both clinical settings and breeding industry by strengthening the detection and analysis of this mobile genetic element.

## Conclusion

In conclusion, increase of cefepime resistance in *S*. Enteritidis is a serious public health concern. Our data demonstrate that the *bla*_CTX–M–55_ gene mobilized by the IS*Ecp1*-*bla*_CTX–M–55_-ORF477 transposition unit is the main feature shared by CRSE and plays an important role in the transmission among different species.

## Data Availability Statement

The data sets generated for this study can be found in the NCBI Sequence Read Archive (SRA) under accession number SRR9734360 (https://www.ncbi.nlm.nih.gov/search/all/?term=SRR9734360).

## Ethics Statements

Ethical approval for this study was provided by Shanghai Municipal Center for Disease Control and Prevention (Shanghai, China). This study is a retrospective study and individual patient identification is not accessed and informed consent is not required.

## Author Contributions

YF, JZ, and ML conceived and designed the experiment. YF and YM carried out the experiment. YF, LZ, ZX, YM, YW, ZC, and JB contributed to sample preparation and collected the data. YF, LZ, ZX, and YM performed the analysis. YF wrote the manuscript. JZ and ML revised the manuscript. XX provided the samples. ML and JZ fund the project. All authors discussed the results and commented on the manuscript.

## Conflict of Interest

The authors declare that the research was conducted in the absence of any commercial or financial relationships that could be construed as a potential conflict of interest.
