# Supplementary material for: Fourth Generation Cephalosporin Resistance Among Salmonella enterica Serovar Enteritidis Isolates in Shanghai, China Conferred by blaCTX–M–55 Harboring Plasmids
Source: Front Microbiol. 2020 May 15;11:910. doi: 10.3389/fmicb.2020.00910 (PMC7242564; doi:10.3389/fmicb.2020.00910)
Supplement: Supplementary file 1 [file Data_Sheet_1.docx]

**Supplementary Information For**

**Fourth generation cephalosporin resistance among *Salmonella* enterica serovar Enteritidis isolates in Shanghai, China conferred by *bla*_CTX-M-55_ harboring plasmid**

Ying Fu^1^, Xuebin Xu^2^, Lina Zhang^1^, Zhiying Xiong^1^, Yeben Ma^1^, Yihuan Wei^1^, Zhengquan Chen^1^, Jie Bai^1^, Ming Liao^1^*and Jianmin Zhang^1^*

*1) Key Laboratory of Veterinary Vaccine Innovation of the Ministry of Agriculture, Key Laboratory of Zoonosis Prevention and Control of Guangdong Province, PR China, College of Veterinary Medicine, South China Agricultural University, Guangzhou 510642, China, 2) Shanghai Municipal Center for Disease Control and Prevention, Shanghai 200336, China*

*Co-corresponding authors: Jianmin Zhang and Ming Liao

Jianmin Zhang, College of Veterinary Medicine, South China Agricultural University, 483 Wushan Road, Tianhe District, Guangzhou 510642, China. E-mail: junfeng-v@163.com;

Ming Liao, College of Veterinary Medicine, South China Agricultural University, 483 Wushan Road, Tianhe District, Guangzhou 510642, China. E-mail: [mliao@scau.edu.cn;](mailto:mliao@scau.edu.cn;)

**Supplementary Table 1.** PCR primers used in this study

| Gene | Prime name | Sequence（5’—3’） | Size | Annealing temperatures | References |
| --- | --- | --- | --- | --- | --- |
| CTX-M | CTX-M-F | GAGTTTCCCCATTCCGTTTC | 909 bp | 50℃ | (Kiratisin et al., 2008; Wu et al., 2015) |
|  | CTX-M-R | CAGAATAAGGAATCCCATGGTT |  |  |  |
| TEM | TEM-F | ATGAGTATTCAACATTTCCG | 964 bp | 50℃ | (Archambault et al., 2006; Wu et al., 2015) |
|  | TEM-R | ACCAATGCTTAATCAGTGAG |  |  |  |
| SHV | SHV-F | TTCGCCTGTGTATTATCTCCCTG | 854 bp | 50℃ | (Archambault et al., 2006) |
|  | SHV-R | TTAGCGTTGCCAGTGCTCG |  |  |  |
| ACC | ACC-F | AGCCTCAGCAGCCGGTTAC | 818 bp | 55℃ | (Archambault et al., 2006) |
|  | ACC-R | GAAGCCGTTAGTTGATCCGG |  |  |  |
| OXA | OXA-F | ACCAGATTCAACTTTCAA | 590 bp | 55℃ | (Usha et al., 2008) |
|  | OXA-R | TCTTGGCTTTTATGCTTG |  |  |  |
| PSE | PSE-F | AATGGCAATCAGCGCTTCCC | 598 bp | 55℃ | (Shahada et al., 2006) |
|  | PSE-R | GGGGCTTGATGCTCACTACA |  |  |  |
| VEB | VEB-F | GATAGGAGTACAGACATATG | 914 bp | 60℃ | (Kiratisin et al., 2008) |
|  | VEB-R | TTTATTCAAATAGTAATTCCACG |  |  |  |
| PER | PER-F | ATGAATGTCATCACAAAATG | 927 bp | 56℃ | (Kiratisin et al., 2008) |
|  | PER-R | TCAATCCGGACTCACT |  |  |  |
| GES | GES-F | ATGCGCTTCATTCACGCAC | 864 bp | 57℃ | (Kiratisin et al., 2008) |
|  | GES-R | CTATTTGTCCGTGCTCAGG |  |  |  |

**Supplementary Table 2.** Drug sensitivity of 38 CRSE to 17 antibiotics

| *Salmonella* isolates | Zone diameters and Breakpoints (mm) | | | | | | | | | | | | | | | | | |
| --- | --- | --- | --- | --- | --- | --- | --- | --- | --- | --- | --- | --- | --- | --- | --- | --- | --- | --- |
|  | AMC | AMP | CTX | CAZ | FEP | IPM | AMK | GEN | STR | SIZ | SXT | PMB | CHL | TET | NAL | CIP | OFX |  |
|  | 18-24 | 16-22 | 29-35 | 25-32 | 31-37 | 26-32 | 19-26 | 19-26 | 12-20 | 15-23 | 23-29 | 13-19 | 21-27 | 18-25 | 22-28 | 30-40 | 29-33 |  |
| SH10G391 | 18 | 0 | 0 | 13 | 13 | 27 | 22 | 22 | 0 | 0 | 20 | 15 | 24 | 23 | 0 | 27 | 20 |  |
| SH11G394 | 18 | 0 | 0 | 14 | 15 | 28 | 23 | 24 | 0 | 0 | 25 | 14 | 27 | 0 | 0 | 30 | 23 |  |
| SH11G405 | 18 | 0 | 0 | 15 | 15 | 30 | 22 | 24 | 0 | 0 | 20 | 14 | 25 | 22 | 0 | 28 | 23 |  |
| SH11G461 | 18 | 0 | 0 | 17 | 15 | 2 | 23 | 24 | 0 | 0 | 0 | 15 | 26 | 0 | 0 | 21 | 15 |  |
| SH11G1338 | 19 | 0 | 0 | 16 | 17 | 28 | 24 | 23 | 0 | 0 | 23 | 15 | 26 | 0 | 0 | 27 | 22 |  |
| SH11G1355 | 20 | 0 | 0 | 19 | 18 | 27 | 23 | 20 | 0 | 0 | 22 | 16 | 29 | 0 | 0 | 26 | 22 |  |
| SH11G1371 | 19 | 0 | 0 | 15 | 14 | 28 | 24 | 23 | 0 | 0 | 24 | 16 | 28 | 0 | 0 | 28 | 21 |  |
| SH12G402 | 17 | 0 | 0 | 20 | 9 | 28 | 23 | 22 | 18 | 16 | 0 | 15 | 27 | 0 | 17 | 27 | 21 |  |
| SH12G460 | 18 | 0 | 0 | 13 | 10 | 29 | 25 | 25 | 0 | 0 | 24 | 16 | 15 | 22 | 0 | 28 | 23 |  |
| SH12G465 | 20 | 0 | 0 | 11 | 11 | 28 | 22 | 20 | 17 | 15 | 24 | 14 | 25 | 22 | 0 | 26 | 20 |  |
| SH12G477 | 18 | 0 | 0 | 15 | 13 | 27 | 20 | 21 | 0 | 0 | 17 | 15 | 22 | 0 | 0 | 23 | 20 |  |
| SH12G514 | 21 | 0 | 0 | 17 | 17 | 27 | 23 | 23 | 0 | 0 | 23 | 15 | 0 | 0 | 0 | 27 | 22 |  |
| SH12G565 | 20 | 0 | 0 | 15 | 17 | 28 | 23 | 23 | 0 | 0 | 23 | 14 | 26 | 0 | 0 | 30 | 23 |  |
| SH12G706 | 18 | 0 | 0 | 15 | 15 | 27 | 21 | 22 | 0 | 0 | 21 | 15 | 27 | 21 | 0 | 27 | 20 |  |
| SH12G729 | 18 | 0 | 0 | 13 | 14 | 30 | 22 | 25 | 23 | 18 | 25 | 15 | 24 | 24 | 0 | 30 | 25 |  |
| SH12G937 | 21 | 0 | 0 | 17 | 12 | 29 | 23 | 28 | 0 | 0 | 20 | 16 | 28 | 0 | 0 | 32 | 27 |  |
| SH12G1019 | 19 | 0 | 0 | 14 | 15 | 27 | 22 | 23 | 0 | 0 | 23 | 14 | 26 | 0 | 0 | 29 | 23 |  |
| SH12G1079 | 23 | 0 | 7 | 18 | 20 | 30 | 22 | 25 | 0 | 0 | 19 | 15 | 0 | 0 | 0 | 30 | 23 |  |
| SH12G1166 | 21 | 0 | 0 | 18 | 16 | 28 | 20 | 21 | 17 | 15 | 21 | 15 | 24 | 19 | 0 | 20 | 18 |  |
| SH12G1178 | 16 | 0 | 0 | 11 | 11 | 29 | 23 | 25 | 0 | 0 | 23 | 15 | 27 | 22 | 0 | 29 | 24 |  |
| SH12G1276 | 17 | 0 | 8 | 21 | 17 | 28 | 23 | 0 | 0 | 0 | 21 | 14 | 26 | 23 | 0 | 31 | 23 |  |
| SH13G474 | 21 | 0 | 0 | 15 | 17 | 30 | 21 | 11 | 20 | 13 | 18 | 15 | 17 | 21 | 0 | 24 | 20 |  |
| SH13G961 | 17 | 0 | 0 | 14 | 15 | 27 | 23 | 22 | 0 | 0 | 23 | 15 | 27 | 22 | 0 | 27 | 22 |  |
| SH13G990 | 21 | 0 | 0 | 17 | 17 | 28 | 22 | 23 | 10 | 16 | 24 | 14 | 0 | 0 | 0 | 27 | 23 |  |
| SH13G1032 | 20 | 0 | 0 | 17 | 14 | 25 | 23 | 23 | 16 | 15 | 24 | 16 | 26 | 21 | 0 | 24 | 22 |  |
| SH13G1838 | 20 | 0 | 0 | 17 | 19 | 29 | 24 | 0 | 21 | 18 | 26 | 16 | 28 | 22 | 24 | 28 | 22 |  |
| SH13G1868 | 19 | 0 | 0 | 16 | 14 | 28 | 18 | 19 | 14 | 14 | 24 | 15 | 27 | 20 | 0 | 23 | 20 |  |
| SH13G1882 | 20 | 0 | 0 | 17 | 17 | 26 | 19 | 18 | 15 | 14 | 18 | 15 | 19 | 21 | 0 | 21 | 17 |  |
| SH13G1958 | 21 | 0 | 0 | 15 | 16 | 28 | 22 | 22 | 0 | 0 | 15 | 15 | 0 | 0 | 0 | 27 | 20 |  |
| SH14G065 | 22 | 0 | 0 | 17 | 17 | 26 | 22 | 20 | 14 | 16 | 23 | 19 | 25 | 20 | 0 | 27 | 19 |  |
| SH14G169 | 21 | 0 | 0 | 17 | 18 | 27 | 20 | 19 | 14 | 0 | 17 | 15 | 19 | 21 | 0 | 22 | 17 |  |
| SH14G548 | 22 | 0 | 0 | 17 | 17 | 28 | 23 | 23 | 19 | 15 | 24 | 14 | 26 | 22 | 0 | 29 | 22 |  |
| SH14G1579 | 21 | 0 | 0 | 16 | 17 | 28 | 23 | 21 | 0 | 0 | 22 | 14 | 7 | 0 | 0 | 28 | 21 |  |
| SH14G1588 | 25 | 0 | 0 | 20 | 21 | 31 | 25 | 25 | 0 | 0 | 23 | 16 | 27 | 21 | 0 | 30 | 22 |  |
| SH14G1019 | 0 | 0 | 0 | 0 | 7 | 15 | 22 | 22 | 13 | 0 | 0 | 16 | 28 | 21 | 0 | 24 | 23 |  |
| SH14G1041 | 0 | 0 | 0 | 0 | 15 | 16 | 25 | 0 | 15 | 0 | 0 | 17 | 28 | 22 | 0 | 28 | 22 |  |
| SH13SF278 | 22 | 0 | 8 | 17 | 17 | 26 | 22 | 20 | 0 | 0 | 17 | 16 | 0 | 0 | 0 | 23 | 20 |  |
| SH14SF008 | 20 | 0 | 0 | 15 | 15 | 25 | 23 | 21 | 0 | 0 | 14 | 16 | 0 | 0 | 0 | 26 | 18 |  |

**Supplementary Table 3.** The resistance pattern of the 38 cefepime-resistant *Salmonella* Enteritidis strains

| The resistance pattern | No. of antibiotics | No. of  resistant strains | Proportion |
| --- | --- | --- | --- |
| AMP-CTX-CAZ-FEP-STR-SIZ-SXT-CHL-TET-NAL | 10 | 5 | 13.16% |
| AMC-AMP-CTX-CAZ-FEP-GEN-STR-SIZ-SXT-NAL | 10 | 1 | 2.63% |
| AMP-CTX-CAZ-FEP-STR-SIZ-SXT-TET-NAL | 9 | 4 | 10.53% |
| AMP-CTX-CAZ-FEP-GEN-SIZ-SXT-CHL-NAL | 9 | 2 | 5.26% |
| AMC-AMP-CTX-CAZ-FEP-GEN-SIZ-SXT-NAL | 9 | 1 | 2.63% |
| AMP-CTX-CAZ-FEP-STR-SIZ-CHL-TET-NAL | 9 | 1 | 2.63% |
| AMP-CTX-CAZ-FEP-STR-SIZ-TET-NAL | 8 | 5 | 13.16% |
| AMP-CTX-CAZ-FEP-STR-SIZ-SXT-NAL | 8 | 3 | 7.89% |
| AMC-AMP-CTX-CAZ-FEP-STR-SIZ-NAL | 8 | 2 | 5.26% |
| AMP-CTX-CAZ-FEP-SIZ-SXT-CHL-NAL | 8 | 1 | 2.63% |
| AMC-AMP-CTX-CAZ-FEP-SXT-TET-NAL | 8 | 1 | 2.63% |
| AMC-AMP-CTX-CAZ-FEP-SIZ-SXT-NAL | 8 | 1 | 2.63% |
| AMP-CTX-CAZ-FEP-STR-SIZ-CHL-NAL | 8 | 1 | 2.63% |
| AMP-CTX-CAZ-FEP-STR-CHL-TET-NAL | 8 | 1 | 2.63% |
| AMP-CTX-CAZ-FEP-AMK-SIZ-NAL | 7 | 1 | 2.63% |
| AMP-CTX-CAZ-FEP-STR-SIZ-NAL | 7 | 1 | 2.63% |
| AMP-CTX-CAZ-FEP-SXT-NAL | 6 | 1 | 2.63% |
| AMP-CTX-CAZ-FEP-GEN | 5 | 5 | 13.16% |
| AMP-CTX-CAZ-FEP-NAL | 5 | 1 | 2.63% |

* AMP: ampicillin, AMC: amoxicillin-clavulanic acid, CTX: cefotaxime, CAZ: ceftazidime, FEP: cefepime, GEN: gentamicin, STR: streptomycin, AMK: [amikacin](C:/Users/rena/AppData/Local/youdao/dict/Application/7.1.0.0421/resultui/dict/?keyword=amikacin), SXT: trimethoprim/sulfamethoxazole, SIZ: sulfisoxazole, NAL: nalidixic acid, OFX: ofloxacin, CIP: ciprofloxacin, CHL: chloramphenicol, IPM: [imipenem](C:/Users/rena/AppData/Local/youdao/dict/Application/7.1.0.0421/resultui/dict/?keyword=imipenem), PMB: polymyxin B, TET: tetracycline

**Supplementary Table 4.** Commonly used drugs susceptibility test for *Salmonella* Enteritidis isolated from Shanghai from 2005-2014

| Antibiotics | Years (n=the number of *S.* Enteritidis isolates) | | | | | | | | | | | |
| --- | --- | --- | --- | --- | --- | --- | --- | --- | --- | --- | --- | --- |
|  | 2005  (n=8) | | 2006  (n=62) | 2007  (n=57) | 2008  (n=125) | 2009  (n=123) | 2010  (n=222) | 2011  (n=679) | 2012  (n=547) | 2013  (n=629) | 2014  (n=462) | Sum  (n=2914) |
| Nalidixic Acid | | 100.0% | 93.5% | 86.0% | 94.4% | 96.7% | 95.0% | 97.9% | 95.8% | 95.7% | 95.7% | 95.1% |
| Sulfisoxazole | | 87.5% | 43.5% | 49.1% | 40.8% | 53.7% | 52.3% | 69.1% | 56.3% | 53.4% | 63.0% | 56.9% |
| Ampicillin | | 25.0% | 25.8% | 35.1% | 38.4% | 56.9% | 58.1% | 64.8% | 62.0% | 65.5% | 70.1% | 50.2% |
| Streptomycin | | 12.5% | 29.0% | 38.6% | 34.4% | 46.3% | 44.6% | 53.0% | 47.5% | 49.6% | 61.3% | 41.7% |
| Tetracycline | | 12.5% | 32.3% | 19.3% | 22.4% | 32.5% | 28.8% | 30.9% | 19.0% | 28.3% | 21.9% | 24.8% |
| Gentamicin | | 0.0% | 9.7% | 5.3% | 5.6% | 11.4% | 16.7% | 10.5% | 10.4% | 4.5% | 2.6% | 7.7% |
| Cefotaxime | | 0.0% | 3.2% | 0.0% | 2.4% | 16.3% | 2.7% | 4.6% | 8.8% | 22.4% | 9.3% | 7.0% |
| Trimethoprim-Sulfamethoxazole | | 0.0% | 6.5% | 1.8% | 5.6% | 11.4% | 12.2% | 10.0% | 7.5% | 5.2% | 4.8% | 6.5% |
| Ceftazidime | | 0.0% | 4.8% | 0.0% | 0.0% | 4.9% | 1.4% | 4.1% | 6.0% | 16.4% | 6.3% | 4.4% |
| Chloramphenicol | | 0.0% | 4.8% | 1.8% | 0.8% | 9.8% | 1.8% | 4.9% | 2.9% | 11.8% | 2.4% | 4.1% |
| Amoxicillin-Clavulanic Acid | | 12.5% | 1.6% | 0.0% | 1.6% | 4.1% | 0.9% | 4.0% | 1.5% | 2.2% | 2.6% | 3.1% |
| Ciprofloxacin | | 0.0% | 1.6% | 0.0% | 0.8% | 4.9% | 1.8% | 2.8% | 0.5% | 1.1% | 0.0% | 1.4% |
| Ofloxacin | | 0.0% | 1.6% | 0.0% | 0.8% | 1.6% | 0.0% | 0.7% | 0.0% | 1.1% | 0.0% | 0.6% |


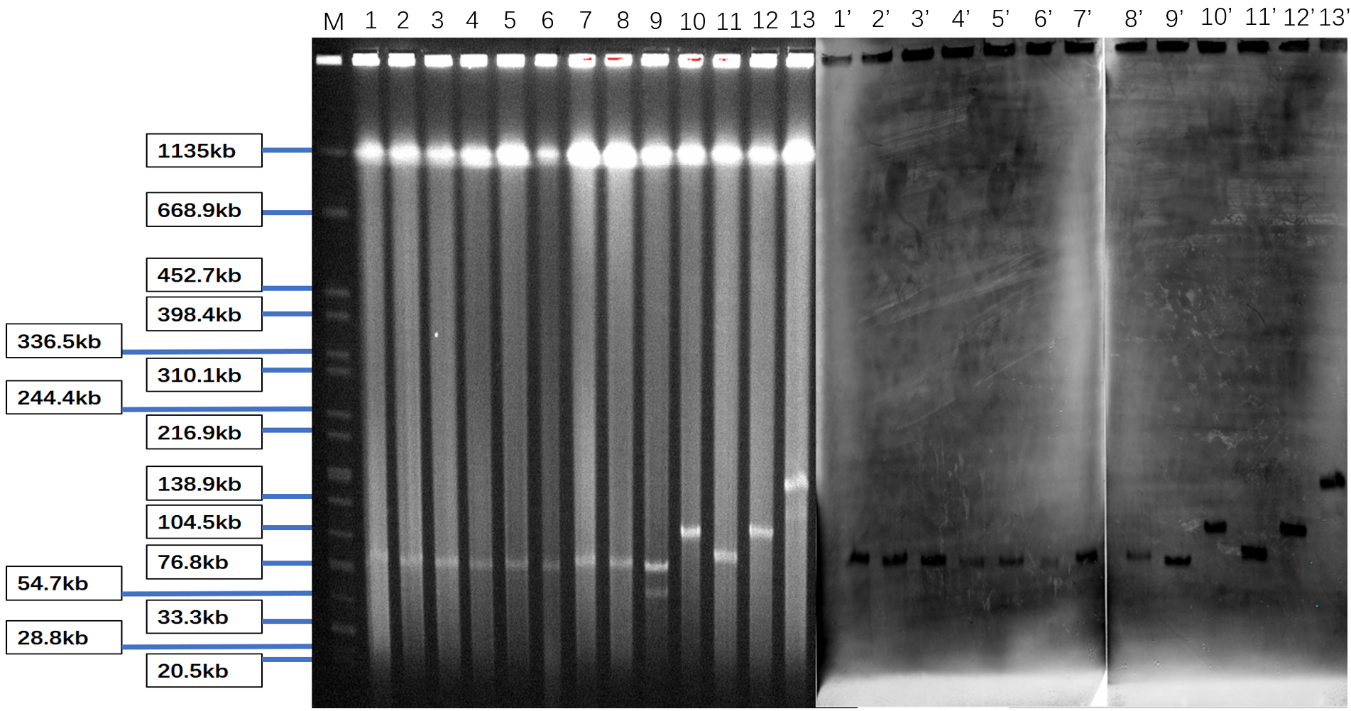


**Fig. S1**. S1-PFGE of cefepime resistant, CTX-M-producing *S.* Enteritidis isolates’ respective transconjugants (left) and Southern blot hybridization with the *bla*_CTX-M_ probe (right). M: H9812, Lanes 1–13: The transconjugants SH11G394-C, SH11G405-C, SH11G1338-C, SH11G1355-C, SH11G1371-C, SH12G565-C, SH12G706-C, SH13G961-C, SH12G460-C, SH12G514-C, SH12G729-C, SH12G1079-C, SH12G1166-C; Lanes 1’–13’: The location of *bla*_CTX-M_ gene by southern blot hybridization with the *bla*_CTX-M_ probe.


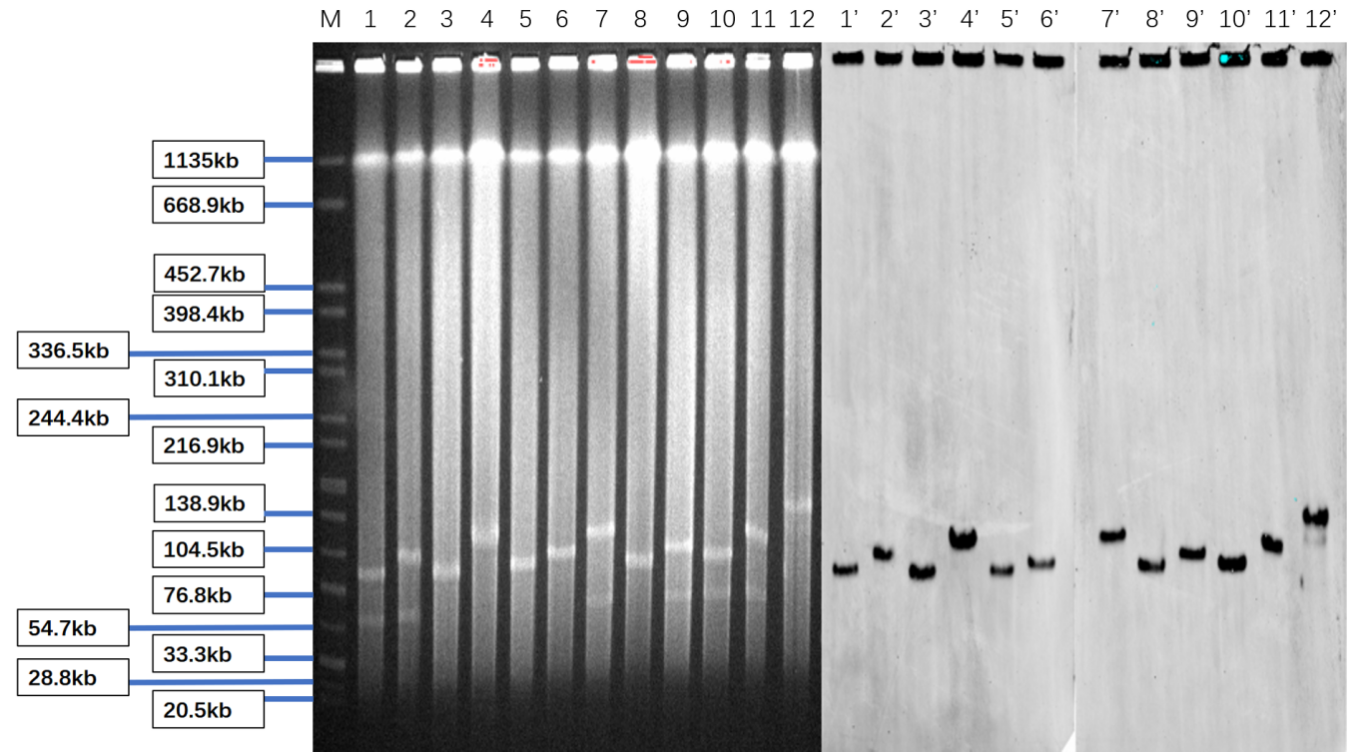


**Fig. S2**. S1-PFGE of cefepime resistant, CTX-M-producing *S.* Enteritidis isolates’ respective transconjugants (left) and Southern blot hybridization with the *bla*_CTX-M_ probe (right). M: H9812, Lanes 1–13: The transconjugants SH13G474-C, SH13G990-C, SH13G1032-C, SH13G1838-C, SH13G1868-C, SH13G1882-C, SH13G1958-C, SH14G065-C, SH14G169-C, SH14G548-C, SH14G1579-C, SH13SF278-C; Lanes 1’–13’: The location of *bla*_CTX-M_ gene by southern blot hybridization with the *bla*_CTX-M_ probe.


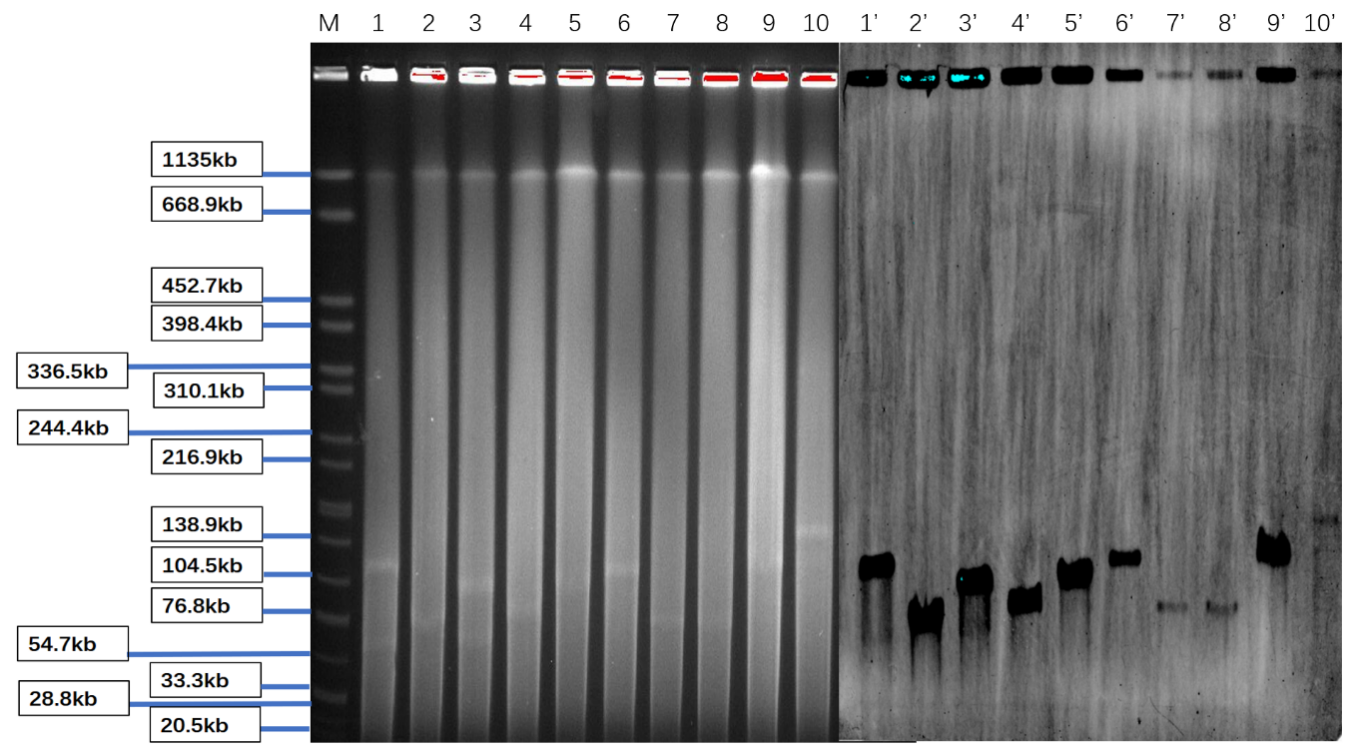


**Fig. S3**. S1-PFGE of cefepime resistant, CTX-M-producing *S.* Enteritidis isolates’ respective transconjugants (left) and Southern blot hybridization with the *bla*_CTX-M_ probe (right). M: H9812, Lanes 1–13: The transconjugants SH14SF008-C, SH12G402-C, SH14G1019-C, SH14G1041-C, SH12G477-C, SH12G1178-C, SH12G465-C, SH12G1019-C, SH10G391-C, SH12G1276-C; Lanes 1’–13’: The location of *bla*_CTX-M_ gene by southern blot hybridization with the *bla*_CTX-M_ probe.

**References**

Archambault, M., Petrov, P., Hendriksen, R.S., Asseva, G., Bangtrakulnonth, A., Hasman, H., et al. (2006). Molecular characterization and occurrence of extended-spectrum beta-lactamase resistance genes among Salmonella enterica serovar Corvallis from Thailand, Bulgaria, and Denmark. Microb Drug Resist 12(3), 192-198. doi: 10.1089/mdr.2006.12.192.

Kiratisin, P., Apisarnthanarak, A., Laesripa, C., and Saifon, P. (2008). Molecular characterization and epidemiology of extended-spectrum-beta-lactamase-producing Escherichia coli and Klebsiella pneumoniae isolates causing health care-associated infection in Thailand, where the CTX-M family is endemic. Antimicrob Agents Chemother 52(8), 2818-2824. doi: 10.1128/AAC.00171-08.

Shahada, F., Chuma, T., Tobata, T., Okamoto, K., Sueyoshi, M., and Takase, K. (2006). Molecular epidemiology of antimicrobial resistance among Salmonella enterica serovar Infantis from poultry in Kagoshima, Japan. Int J Antimicrob Agents 28(4), 302-307. doi: 10.1016/j.ijantimicag.2006.07.003.

Usha, G., Chunderika, M., Prashini, M., Willem, S.A., and Yusuf, E.S. (2008). Characterization of extended-spectrum beta-lactamases in Salmonella spp. at a tertiary hospital in Durban, South Africa. Diagn Microbiol Infect Dis 62(1), 86-91. doi: 10.1016/j.diagmicrobio.2008.04.014.

Wu, H., Wang, Y., Wu, Y., Qiao, J., Li, H., Zheng, S., et al. (2015). Emergence of beta-lactamases and extended-spectrum beta-lactamases (ESBLs) producing Salmonella in retail raw chicken in China. Foodborne Pathog Dis 12(3), 228-234. doi: 10.1089/fpd.2014.1859.
